# Development of Terminator–Promoter Bifunctional Elements for Application in *Saccharomyces cerevisiae* Pathway Engineering

**DOI:** 10.3390/ijms24129870

**Published:** 2023-06-07

**Authors:** Xiaoxia Ni, Zhengyang Liu, Jintang Guo, Genlin Zhang

**Affiliations:** Key Laboratory for Green Processing of Chemical Engineering of Xinjiang Bingtuan, School of Chemistry and Chemical Engineering, Shihezi University, Shihezi 832003, China

**Keywords:** synthetic regulatory element, pathway engineering, promoter, terminator, transcriptional regulation

## Abstract

The construction of a genetic circuit requires the substitution and redesign of different promoters and terminators. The assembly efficiency of exogenous pathways will also decrease significantly when the number of regulatory elements and genes is increased. We speculated that a novel bifunctional element with promoter and terminator functions could be created via the fusion of a termination signal with a promoter sequence. In this study, the elements from a *Saccharomyces cerevisiae* promoter and terminator were employed to design a synthetic bifunctional element. The promoter strength of the synthetic element is apparently regulated through a spacer sequence and an upstream activating sequence (UAS) with a ~5-fold increase, and the terminator strength could be finely regulated by the efficiency element, with a ~5-fold increase. Furthermore, the use of a TATA box-like sequence resulted in the adequate execution of both functions of the TATA box and the efficiency element. By regulating the TATA box-like sequence, UAS, and spacer sequence, the strengths of the promoter-like and terminator-like bifunctional elements were optimally fine-tuned with ~8-fold and ~7-fold increases, respectively. The application of bifunctional elements in the lycopene biosynthetic pathway showed an improved pathway assembly efficiency and higher lycopene yield. The designed bifunctional elements effectively simplified pathway construction and can serve as a useful toolbox for yeast synthetic biology.

## 1. Introduction

A key concept in synthetic biology is the development of standardized multifunctional components [1]. Specifically, structurally different elements have the potential to develop new properties in new combinatorial structures, which will result in the creation of novel genetic elements [2]. Many natural gene elements, such as promoters and terminators*,* are essential transcriptional regulators of gene expression and pathway engineering that exhibit excellent structural properties and whose functions are apparent [3,4,5,6]. With the improvement in natural gene elements, synthetic genetic elements have the advantage of a streamlined structure for easy regulation and a controlled synthesis length, with minimal sequence homology and better performance. Generally, random or rational construction methods have been widely used to develop novel synthetic gene elements, which have been successfully applied to the expression of metabolic engineering-related enzymes in recent decades. As the most basic functional element in metabolic engineering, promoters are the most effective tool for regulating metabolic pathways at the transcriptional level. By analyzing the structure of eukaryotic promoters, Juven et al. [7] described the design of a super core promoter (S_CP_) to enhance gene expression, which contained a TATA box, initiator (Inr), motif ten element (MTE), and downstream promoter element (DPE). This promoter directs high levels of transcription by RNA polymerase II in eukaryotic cells. The copy number of the UAS was found to be another important factor. The intensity of the promoter could be increased 400 times by changing the copy number of the UAS [8]. Sen et al. [9] constructed a broad-spectrum promoter by integrating the core promoter regions of *E. coli*, *S. cerevisiae*, and *B. subtilis*, which demonstrated normal function in a range of different hosts.

Transcription terminators are another important element for regulating gene expression in synthetic gene networks. The terminator sequence in eukaryotic genes are located in the 3′ untranslated region, and its activity is directly related to mRNA stability, and thus the regulation of gene expression [10]. Guo et al. [5,11] found that a yeast terminator usually consists of three elements: an efficiency element, a positioning element, and a poly(A) site. In addition, linker 1 (situated between the efficiency and positioning elements) and linker 2 (between the positioning element and the poly(A) site) can finely regulate the intensity of the terminator [12]. Considering the transcription process, it begins at the promoter and terminates at a particular RNA sequence encoded by the DNA terminator, thereby affecting a particular pause in RNA polymerase (RNAP) with ultimate breakdown of the transcriptional complex to release the RNA product. Kang et.al [13] reported that the population of RNA polymerase that bound to the intrinsic terminator usually releases RNA transcription products without departing from the DNA template, and the remaining RNA polymerase molecules diffuse one-dimensionally in both directions on DNA, even reinitiating transcription at nearby promoters. 

The assembly of regulatory elements and genes is a basic procedure. However, in yeast, standard parts, such as promoters and terminators, are repeatedly used for genetic circuit construction, which may lead to the rearrangement of repetitive sequences in multigene assemblies [14,15,16,17,18]. This problem is a major obstacle when assembling complex synthetic circuits or pathways. Moreover, as the number of assembled fragments increases, the efficiency of DNA ligation decreases [2,19,20]. DNA assembly involving multiple fragments is the most time-consuming, and the cost of quality control will also increase. Therefore, the need for highly robust and efficient multifunctional regulatory elements has become an important direction for synthetic biology research. 

In this study, we thus aimed to establish a regulatory element capable of simultaneously regulating the expression of two genes in yeast. That is, an element in a gene circuit not only performs the function of transcriptional termination of upstream genes but also possesses the ability to recruit RNA polymerase II to induce transcription by acting on the promoters of downstream genes. The terminator–promoter bifunctional element simplifies the construction process in gene circuits and pathway engineering, serving as a useful toolbox for the development of yeast metabolic engineering.

## 2. Results and Discussion

### 2.1. Design of Synthetic Bifunctional Element

**Figure 1:**
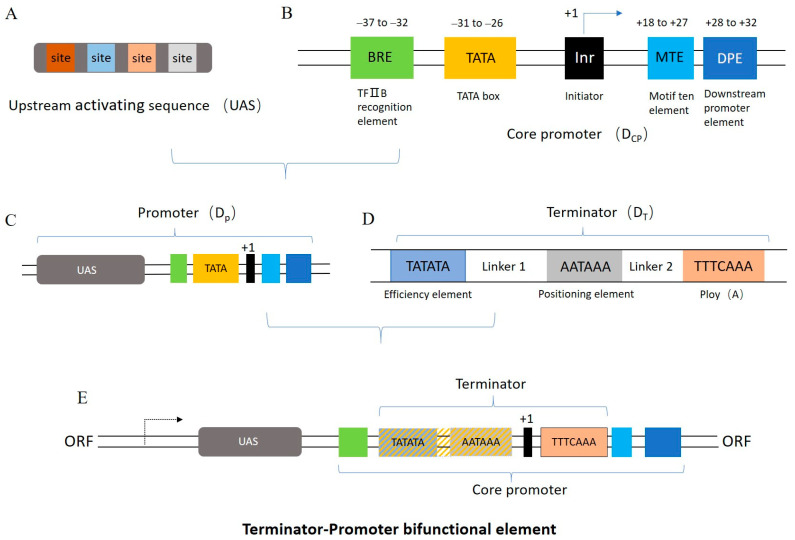
Design of functional elements. (**A**) Structural sketch of upstream activating sequences. The transcription factor binding sites (TFBSs) contain Rap1, Gcr1, Mig1, and Gcr2. Color code does not refer specifically to a site. (**B**) Schematic design of yeast core promoter D_CP_. (**C**) Complete promoter element D_P_ was formed by combining UAS and D_CP_. (**D**) Terminator element D_T_ composed of efficiency element 5′-TATATA-3′, positioning element 5′-AATAAA-3′, and Poly(A). (**E**) Schematic diagram of the terminator–promoter bifunctional element Bif-b1, where terminator sequences are fused into promoter sequences. That is, orange stripes were added to the blue and gray rectangles to indicate that these elements have now acquired the functionality of a TATA box. This is one of several fusion methods, which we discuss in detail in Section 2.4.

The basic sequences for the promoter and terminator were used to build the synthetic bifunctional element with terminator and promoter functions. First, sequences that may help the basal transcription for the core promoter were determined. In *S. cerevisiae*, TATATAA is recognized as being the optimal binding site sequence for recognition by the TATA-box binding protein (TBP) and is conserved [21]. The finest conserved sequence of the TFIIB recognition element (BRE) is GGACGCC, which is adjacent to the TATA box and synergistic with the TATA box [22,23]. The initiator (Inr), which contains the transcription start site, mainly recruits the cofactors and assembles a preinitiation complex (PIC) consisting of Pol II and general transcription factors (GTFs), such as TBP, TFIID, and TFIIB [24]. The conservative DPE sequence is GACGTGC, which can serve as a binding site for transcription factor TFIID by cooperating with the Inr element when a TATA box is lacking [25]. The conserved MTE sequence, with a strong synergistic effect between the TATA box and DPE, is GAGCCGAGCA [26]. Based on these elements, a core promoter, called D_CP_, was assembled by connecting the BRE, TATA box, Inr, MTE, and DPE via linker sequences. Additionally, an upstream activating sequence (UAS) that contains yeast transcription factor binding sites was designed and added upstream of the core promoter D_CP_ (Figure 1A,B) [4]. The UAS can help the core promoter to recruit transcription factors and RNA polymerase II for transcription initiation, thereby enhancing gene expression or achieving specific regulation [6,27,28]. The combination of the UAS and D_CP_ formed a synthetic promoter named D_P_ (Figure 1C).

Previous studies have shown that a terminator implementing transcriptional termination requires at least three basic elements: an efficiency element, a positioning element, and a poly(A) site [11]. The sequence TATATA, as an efficiency element, is considered to be the strongest termination signal [29]. The positioning element is regarded as essential for proper 3′ end formation, as well as cleavage and polyadenylation, and the optimal sequence is AATAAA [30,31]. The poly(A) site plays a role in mRNA transcription and translation, and the optimal sequence is TTTCAAA [11,32]. The sequential ligation of TATATA, linker 1, AATAAA, linker 2, and TTTCAAA produced a synthetic terminator (D_T_) (Figure 1D). After determining the sequences of the promoter and terminator, their different elements were aligned and fused to produce a synthetic element with promoter and terminator functions, and the overall construct is referred to as the terminator–promoter bifunctional element (Figure 1E, Table S1).

### 2.2. Regulation of Promoter Activity by Spacer Sequence and UAS

The synthetic promoter consisting of a core promoter region and UAS was designed to guide the expression of green fluorescent protein in plasmid pRS-Exp1 with CYC1t as a terminator to indirectly characterize its activity in terms of fluorescence intensity (Figure S1A). Conserved regions of the core promoter are generally not specifically regulated; therefore, the mutation of spacer sequences is a common method of engineering for regulating gene expression [1,33]. By altering the spacer sequences, even the binding of a promoter to specific activators to increase promoter activity by several orders of magnitude can be achieved [34]. 

**Figure 2:**
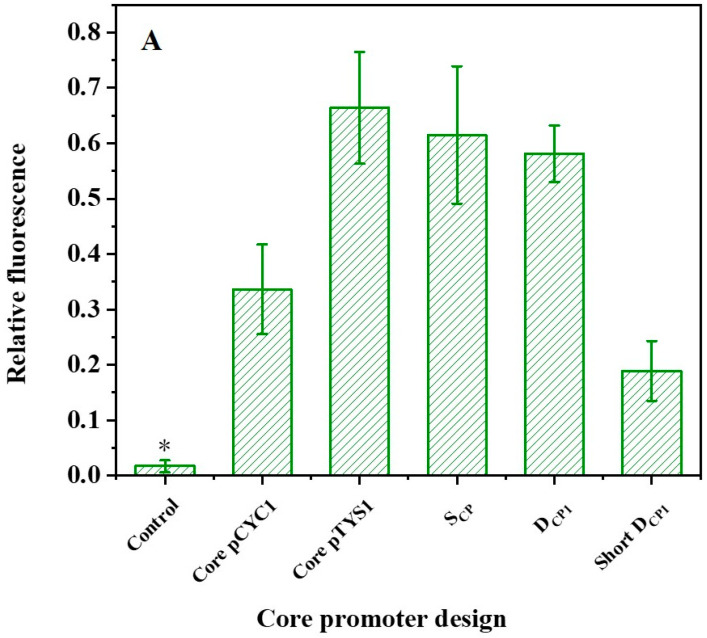

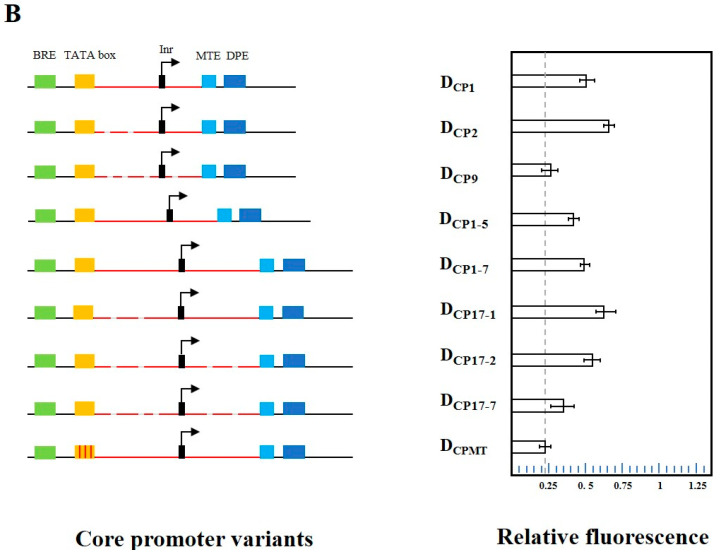
Regulation of promoter activity. (**A**) The activities of designed core promoters. * The fluorescence levels are not statistically significantly different from the blank control. (**B**) Effect of non-conserved regions and TATA box on core promoter activity. The structure of the core promoter variants in the left panel corresponds to the activity presented in the right panel. Red line indicates a non-conserved region. The different lengths represent variations in the length of the spacer sequence, and the breakpoints represent mutations. Compared to D_CP1_, the non-conserved regions of D_CP2_ and D_CP9_ have the same spacer sequence length but different mutations, D_CP1-5_ and D_CP1-7_ both have different spacer sequence lengths; and D_CP17-1_, D_CP17-2_, and D_CP17-3_ have different spacer sequence lengths and different mutations. The TATA box sequence of the mutant D_CPMT_ is mutated to GAGAGA. The left area of the gray line indicates relative fluorescence values below 0.25, defined as non-expression, i.e., no statistical difference relative to the negative control. Data represent the mean fluorescence intensity with error bars (standard error of the mean; SEM) from three independent experiments.

The transcriptional function of the core promoter element D_CP1_, consisting of the BRE element (located at −37 to −32 bp of the transcription start site), TATA box (−31 to −26 bp), Inr (+1), MTE (+18 to +27 bp), and DPE (+28 to +32 bp), was first tested. In order to simplify the sequence composition, a short D_CP1_ was also designed by shortening the spacer sequence between the D_CP1_ elements to 5 bp. The super core promoter (S_CP_) and the core promoter regions of the natural promoters CYC1 and TYS1 were used as positive controls to verify the basic transcriptional ability of the core promoter element [7]. The control indicates the relative fluorescence values yielded by the transformation of the nonmodified pRS-Exp1 in *S. cerevisiae*. The results show that the core promoter designed in this study successfully promotes expression of the fluorescent protein. Compared with the native core promoter TYS1, the expression of D_CP1_ reaches 88%. However, the fluorescence intensity of short D_CP1_ decreased by 67%, indicating that the length of the spacer sequence appears to affect the intensity of the core promoter element (Figure 2A).

Furthermore, we designed five spacer sequences with different mutation regions to test the effect of non-conserved sequences on promoter activity. As shown in Figure 2B, mutations in non-conserved regions can achieve about 0.5~1.3-fold intensity. The negative control lacking the promoter region in the expression cassette was used as a threshold to assess whether the synthesized core promoter is active. In this assay, synthetic core promoters with relative fluorescence values below 0.25 are considered nonfunctional. Moreover, studies have shown that mutations in the TATA box region also affect the promoter strength [35,36]. In parallel, our results show that the mutant D_CPMT_ lacks transcriptional activity. Apparently, the TATA box is an important promoter element. 

**Figure 3:**
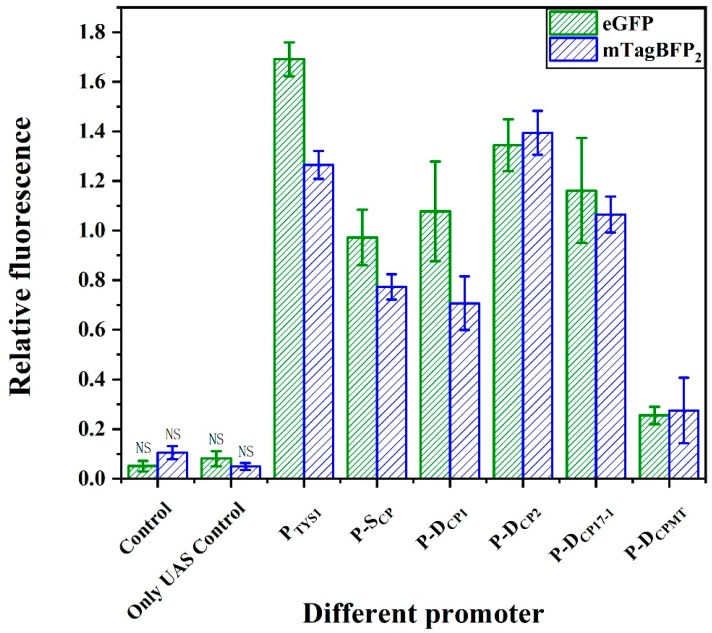
Activity of synthetic promoters consisting of UAS and different core promoters. P-D_CPn_: the UAS assembled with D_CPn_ to form different promoters. Among them, P-D_CP2_ showed the highest activity. Mean fluorescence intensity ± SEM values from triplicate experiments are shown. NS: Not Significant. The fluorescence levels are not statistically significantly different from the blank control.

Transcription factors Rap1, Gcr1, Gcr2, and Mig1 are thought to most effectively influence RNA polymerase II transcription initiation [37,38]. To increase the efficiency of RNA polymerase II binding to the promoter, the UAS region encompassing the binding sites for Rap1, Gcr1, Mig1, and Gcr2 was designed. Their corresponding binding sites were sequentially linked by 20 bp random sequences, which were designed using the R2oDNA Designer website to ensure that no yeast proteins or transcription factor binding sites were included [39]. The assembly of UAS_1_ to the 5′ end of the core promoter element D_CP1_ formed a complete promoter P-D_CP1_. This design resulted in an 86% increase in the fluorescent protein expression compared with D_CP1_, and its intensity reached 64% of the native promoter P_TYS1_ (Figure 3). Similarly, the UAS_1_ also enhanced the activities of the promoters P-D_CP2_ and P-D_CP17-1_, which were increased by 107% and 136% compared with D_CP2_ and D_CP17-1_, respectively. However, it is worth mentioning that when UAS_1_ is added to the 5′ end of D_CPMT_ (P-D_CPMT_), it failed to increase the expression. This may because the mutations in the TATA box resulted in it no longer being recognized by RNA polymerase II (Figure 3). To verify the reproducibility of this result, we constructed another validation vector, pRS-Exp5 with blue fluorescent protein mTagBFP_2_ as a reporter (Figure S1E), and observed similar results. 

Considering the modularity verified in the system, and allowing for sufficient spacing between the UAS and RNA polymerase II binding sequences, the determined core promoter–UAS spacer was maintained in all subsequent core promoter substitutions. That is, the core promoter spacing was set to 20 bp upstream of the BRE element. In another negative control, there was a complete loss of function when the core promoter was removed (only in the UAS control), which confirms that the UAS-only sequence is unable to initiate transcription. 

### 2.3. Regulation of mRNA and Fluorescent Reporter Protein Levels by the Synthetic Terminator Elements

**Figure 4:**
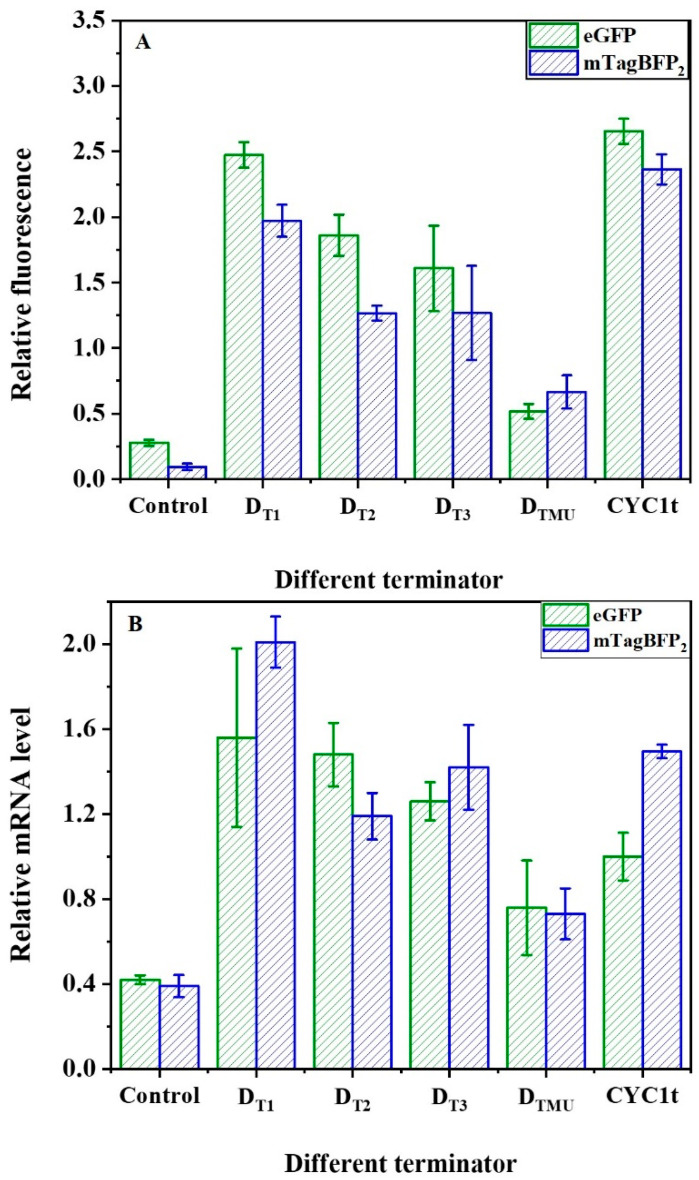
Design of synthetic terminators (**A**) Effects of efficiency element on synthetic terminator (D_Tn)_ strength. Data represent the mean fluorescence intensity with error bars (standard error of the mean (SEM)) from three independent experiments. (**B**) The relative mRNA of terminators. Relative mRNA level was calculated based on the mRNA level of the reference gene *TUB1*. Error bars indicate standard deviation (SD). Data are means: SD (*n* = 3).

The vectors pRS-Exp2 with eGFP and pRS-Exp4 with mTagBFP2 were constructed (Figure S1B,D) to verify the regulation of synthetic terminator elements on mRNA and fluorescent protein levels. The natural strong promoter P_TYS1_ was used to control the transcriptional initiation of fluorescent proteins [40,41,42]. The terminator can significantly regulate gene expression in bacterial and fungal systems and human cells [43,44,45]. The selection of the terminators for heterologous expression can result in desirable or maximum protein yields in yeast. The minimal synthetic sequence required to achieve the terminator function has been demonstrated in previous studies [11]. In particular, the efficiency element is located upstream of the terminator sequence and is an important element affecting its activity [11,29]. Thus, terminators D_T1_, D_T2_, and D_T3_ were constructed with the efficiency elements TATATA, TACCTA, and TATGTC, respectively. Furthermore, because the linker 1 and linker 2 sequences also have a notable effect on its activity, the linker sequences TCCCTTTTCT and GAAAGGGC for the strongest terminator were used [12]. The D_T1_ with efficiency element TATATA demonstrated the same relative fluorescence levels as the natural terminator CYC1 (Figure 4A). Further, the relative mRNA of the fluorescent proteins controlled by different terminators designed in this paper was calculated based on the mRNA of the reference gene *TUB1* (as shown in Figure 4B). The transcription levels and fluorescence values of fluorescent proteins showed similar trends. Therefore, the synthetic terminators in this study could regulate the fluorescent protein levels. When the efficiency element TATATA was replaced with TACCTA or TATGTC, the relative fluorescence decreased by 25% (D_T2_) or 35% (D_T3_) relative to that of D_T1_. Moreover, if the efficiency element TATATA is mutated to GAGAGA (D_TMU_), the relative fluorescence decreases significantly to only 21% that of D_T1_. Similarly, the vector pRS-Exp4 with blue fluorescent protein mTagBFP_2_ was applied to verify the fluorescent reporter protein levels with similar results. In short, the transcription termination function of the synthetic terminators can be finely regulated by the efficiency element.

### 2.4. TATA Box-like Sequence Can Execute Both Functions of TATA Box and Efficiency Element

**Figure 5:**
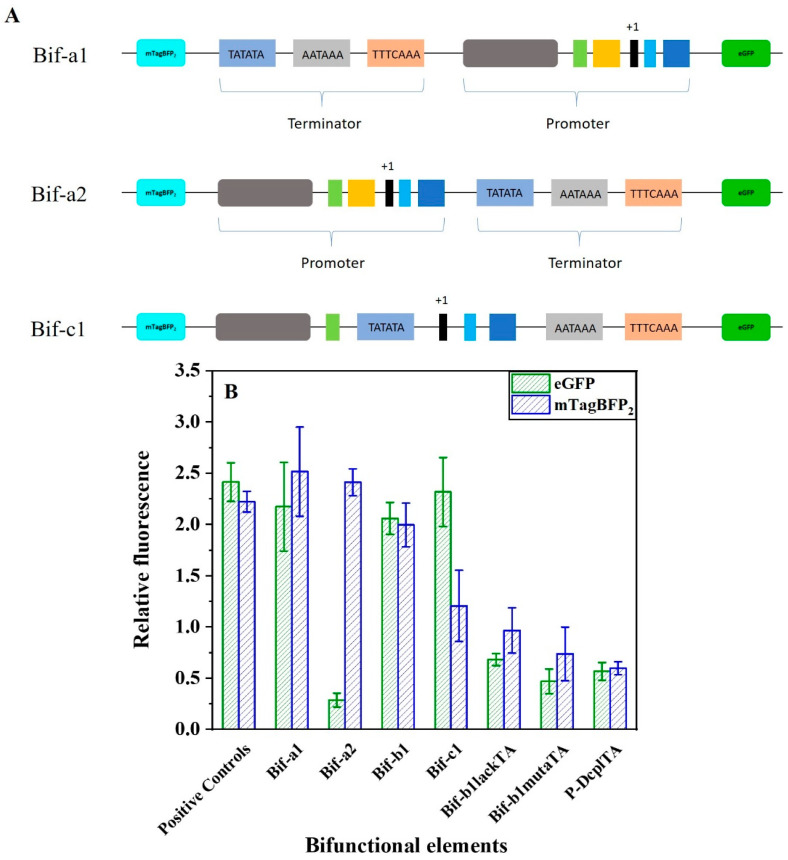
Design and activity characterization of bifunctional elements. (**A**) A schematic of different designs for constructing the bifunctional elements. Their activities are characterized in the reporter plasmid pRS-Exp3, where mTagBFP was inserted upstream and eGFP downstream (**B**) Characterization of the activities of bifunctional elements. The relative FI of mTagBFP2 indirectly indicates the terminator-like strength of bifunctional element. For the eGFP, its relative FI indicates the promoter-like strength of bifunctional elements. Data represent the mean fluorescence intensity with error bars (standard error of the mean; SEM) from three independent experiments.

To construct a regulatory element capable of simultaneously regulating the expressions of two genes in yeast, we fused the strong promoter P-D_CP2_ and the strong terminator D_T1_. The direct assembly of D_T1_ and P-D_CP2_ generated new elements Bif-a1 (D_T1_-P-D_CP2_) or Bif-a2 (P-D_CP2_-D_T1_). For functional validation, pRS-Exp3 was also constructed (Figure S1C). In the vector pRS-Exp3, the natural terminator ADH1 and promoter FBA1 were selected as positive controls to regulate the transcription terminator of the upstream gene and the promoter of the downstream gene. The terminator-like strength and promoter-like strength were characterized by the relative fluorescence values of the blue fluorescent protein (mTagBFP2) and green fluorescent protein (eGFP), respectively. As shown in Figure 5, the terminator-like strength of the bifunctional element Bif-a1 was elevated by 11% compared to the natural terminator ADH1, and its promoter-like strength reached 90% of the promoter FBA1. However, the terminator-like and promoter-like strength of the bifunctional element Bif-a2 increased by only 9% and reached only 12% of the natural promoter FBA1. The results show that the assembly mode of the terminator–promoter achieved the expected functions (Figure S2). The combined mode of terminator–promoter caused the promoter insulation, failing to transcribe the downstream gene [46]. The combination mode of the terminator–promoter is no different from the conventional regulatory elements. Therefore, we attempted to insert the termination signal inside the promoter, not only to perform the transcriptional termination on upstream genes but also to regulate the expressions of downstream genes by recruiting RNA polymerase II. 

Considering the high homology of the TATA box with the efficiency element, we replaced the TATA box in Bif-a2 with the efficiency element to form the Bif-c1. (Figure 5A). The result of the activity verification showed that the terminator-like strength was significantly reduced. We speculated that the spacing of the efficiency element and the positioning element would have a significant effect on the terminator strength. Thus, the terminator D_T1_ was used to replace the TATA box to construct a novel regulatory element, Bif-b1 (Figure 1E). As shown in Figure 5B, the Bif-b1 successfully regulated the expressions of fluorescent proteins in *S. cerevisiae*. In short, the efficiency element and the positioning element combine to perform the function of a TATA box in a bifunctional element. To further confirm the essential TATA sequence in the bifunctional element, we constructed Bif-b1lackTA through the lack of a TATA box and Bif-b1mutaTA through a mutant of the TATA box (Figure S2). Compared to Bif-b1, the activities of the promoter-like and terminator-like Bif-b1lackTA were decreased by 67% and 52%, and those of Bif-b1mutaTA were decreased by 77% and 63%, respectively. These results support the hypothesis that a TATA box-like efficiency element is capable of recruiting RNA polymerase II and initiating the mRNA transcription. 

We used a de novo design approach to synthesize promoter and terminator sequences in this study. The design was based on the structural analysis of eukaryotic transcription initiation and transcriptional termination to find the required motif. Synthetic promoters and terminators were first tested experimentally in the yeast. However, problems were caused by the multiple gene assemblies in the construction of engineered strains of *Saccharomyces cerevisiae,* mainly including sequence duplication rearrangement, time-consuming assembly, and inefficiency. In terms of genetic elements, the regulation of gene expression in yeast is altered only through promoters or terminators. One element with the two functions of terminator and promoter has not yet been studied. Therefore, we are interested in developing novel regulatory elements. We based the fusion on the sequences of a synthetic promoter and a synthetic terminator. As shown in Figure 5, the design approach of Bif-b1 (Figure 1E) for developing a sequence of elements with two functions was ultimately successful.

### 2.5. Fine-Tuning the Activities of the Bifunctional Element

**Figure 6:**
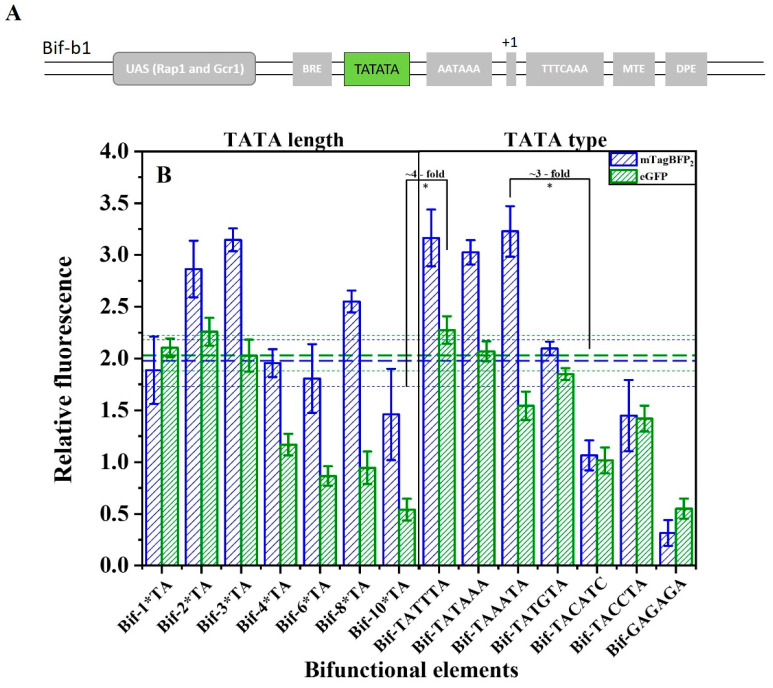
(**A**) Schematic representation of TATA sequence to regulate the activity of bifunctional elements. (**B**) The effect of the number of TATA repeats and type on the activity of the bifunctional element. n*TA indicates the number of “TATA” repeats. Horizontal green bold dashed line indicates the activity of promoter-like of the Bif-b1, and the green dashed line represents the standard error. Horizontal blue bold dashed line indicates the terminator-like activity of the Bif-b1, and the blue dashed line represents the standard error. Data represent the mean fluorescence intensity with error bars (standard error of the mean; SEM) from three independent experiments. Asterisks * indicate a significant difference in the strength of elements in the library (* *p* ≤ 0.05).

To meet the requirements of finely regulating a genetic circuit or biosynthetic pathway, the activity of the terminator–promoter bifunctional element was further adjusted based on numerous considerations. Considering the significant effect of the efficiency element and TATA sequence on the terminator and promoter activity, and the peculiarity of the bifunctional element sharing a common TATA sequence [47,48], we first regulated the activity of the bifunctional element based on repetition and the type of TATA. Based on Bif-b1, a small bifunctional element library was constructed. Figure 6 shows that the terminator-like strength (mTagBFP2 expression) reaches the highest level when TATA is tripled. The promoter-like strength (eGFP expression) is the highest at 1*TA–3*TA. However, if there are more than three TATA repeats, the strength of the bifunctional elements decreases significantly. When the TATA sequence was mutated to other different types, the strength of the bifunctional elements decreases with the decreasing homology of the TATA type with TATTTA. By changing the length and type of TATA, the terminator-like strength of the bifunctional elements was modulated to as high as ~3-fold, and its promoter-like strength was modulated to as high as ~4-fold. 

**Figure 7:**
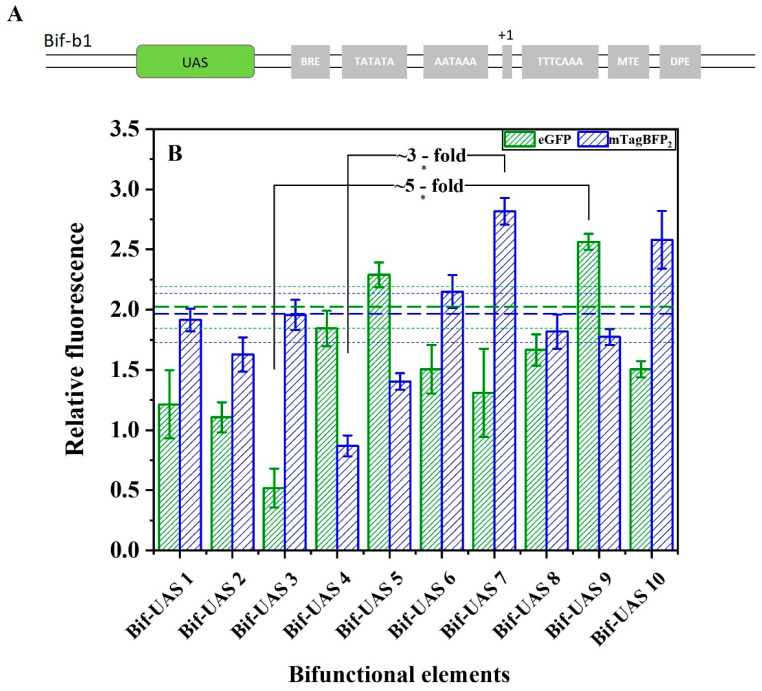
Effect of UAS on the activity of the bifunctional element. (**A**) Schematic representation of UAS to regulate the activity of bifunctional elements. (**B**) UAS1–UAS10 indicate different combinations of Rap1, Gcr1, Mig1, and Gcr2 in the bifunctional element. Horizontal green bold dashed line indicates the activity of promoter-like of the Bif-b1, and the green dashed line represents the standard error. Horizontal blue bold dashed line indicates the terminator-like activity of the Bif-b1, and the blue dashed line represents the standard error. Data represent the mean fluorescence intensity with error bars (standard error of the mean; SEM) from three independent experiments. Asterisks * indicate a significant difference in the strength of elements in the library (* *p* ≤ 0.05).

Because a tandem copy of the upstream active sequence (UAS) can act as a synthetic transcriptional amplifier to increase promoter activity [8], a second attempt was made to finely regulate transcriptional initiation of the bifunctional elements. The different combinations of the transcription factor binding sites (TFBSs: Rap1, Gcr1, Mig1, and Gcr2) shown in Table S2 were linked to Bif-b1, generating another bifunctional element library (Table S3). As shown in Figure 7, the promoter-like strength of Bif-UAS5 and Bif-UAS9 reached 11% and 25% that of Bif-b1. The terminator-like strength of Bif-UAS7 and Bif-UAS10 reached 41% and 29% that of Bif-b1. The modification of the UAS achieved the highest increases of ~5-fold and ~3-fold for the promoter-like and terminator-like strength of bifunctional elements, respectively.

**Figure 8:**
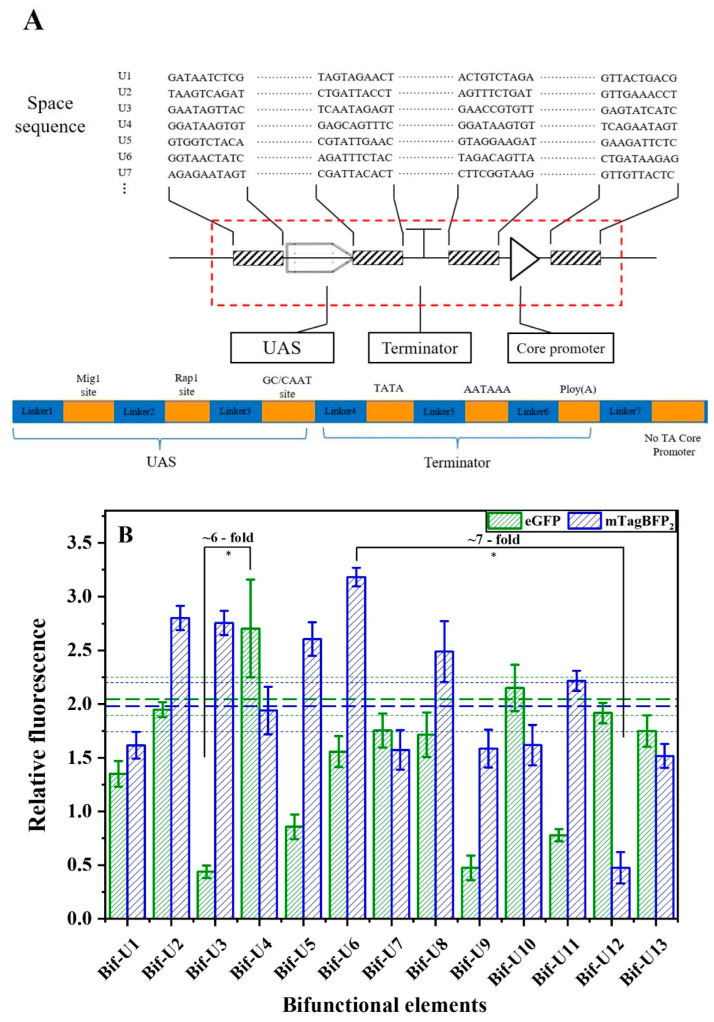
Effect of spacer sequence on the activity of bifunctional element. (**A**) Schematic diagram of different spacer sequences inserted into bifunctional element. Blue box shows the spacer sequence designed by R2oDNA designer. (**B**) Activity of bifunctional elements containing different spacer sequences. Control indicates transformation of pRS-Exp3 plasmid, without any alteration, into yeast in vivo. Horizontal green bold dashed line indicates the promoter-like activity of the Bif-b1, and the green dashed line represents the standard error. Horizontal blue bold dashed line indicates the activity of terminator-like of the Bif-b1, and the blue dashed line represents the standard error. Data represent the mean fluorescence intensity with error bars (standard error of the mean; SEM) from three independent experiments. Asterisks * indicate a significant difference in the strength of elements in the library (* *p* ≤ 0.05).

In addition, to suppress undesirable homologous recombination events that are present in genetic circuits in yeast, another library was constructed by inserting different spacer sequences between the individual motifs of the bifunctional element (Figure 8A). The relative fluorescence values show that the spacer sequences designed by the R2oDNA designer could increase the terminator-like strength by ~7-fold and promoter-like strength by ~6-fold (Figure 8B). These results show that the tuning of bifunctional element strength can be achieved by varying the spacer sequences. Specifically, compared with Bif-b1, the promoter-like strength of U4 was increased by 31%, and the terminator-like strength of U6 was increased by 59%. Repeated sequences may be prone to recombination; thus, spacer sequences could be advantageous to the construction of genetic circuits and the fine regulation of pathways [17]. In short, through the abovementioned construction and regulation, the design of flexible bifunctional elements can also be achieved. The promoter-like strength of bifunctional elements was increased by 8-fold, and its terminator-like strength was increased by 7-fold.

### 2.6. Application of the Bifunctional Element in Pathway Engineering

**Figure 9:**
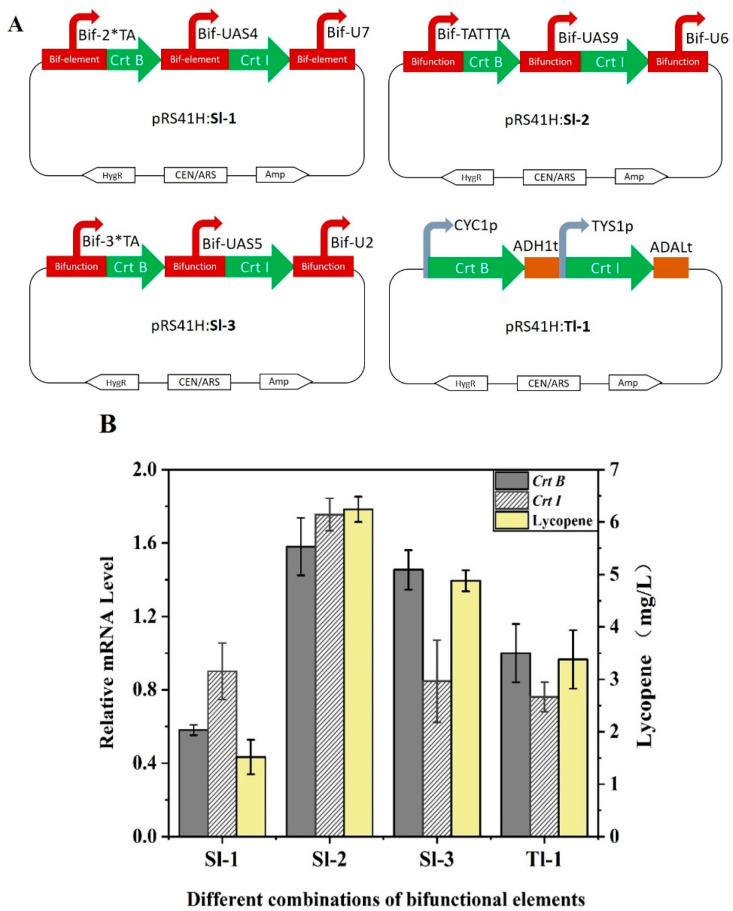
Application of bifunctional elements in lycopene synthesis pathway. (**A**) The detailed genotype of engineered strains. Strains Sl-1, Sl-2 and Sl-3 were constructed using bifunctional elements. Strain Tl-1 was obtained using the native promoters and terminators. (**B**) Lycopene production and relative mRNA with engineered yeasts. Relative mRNA levels were calculated based on the mRNA level of the reference gene *TUB1*. Error bars indicate ± SD. Data are means ± SD (*n* = 3).

As a proof of concept, the lycopene synthesis pathway based on the synthetic bifunctional elements was constructed in *S. cerevisiae.* Because yeast contains the MVA pathway to produce the intermediate product geranylgeranyl diphosphate (GGPP) for lycopene synthesis, exogenous phytoene synthase *Crt B* and phytoene desaturase *Crt I* need to be introduced to *S. cerevisiae* [49]. To verify the function of bifunctional elements in pathway engineering, plasmid-based strains were constructed by transforming gene expression cassettes pRS—Bif-2*TA—*Crt B*—Bif-UAS4—*Crt I*—Bif-U7, pRS—Bif-TATTTA—*Crt B*—Bif-UAS9—*Crt I*—Bif-U6, and pRS—Bif-3*TA—*Crt B*—Bif-UAS5—*Crt I*—Bif-U2 into *S. cerevisiae* dubbed as Sl-1, Sl-2 and Sl-3, respectively (Figure 9A). Strain Tl-1 was a control strain constructed by transforming the expression cassette pRS—CYC1p—*Crt B*—ADH1—TYS1—*Crt I*—ADAL. According to the results of HPLC analysis, lycopene was synthesized by all engineered strains, and the titer of lycopene increased by 85% in strain Sl-2 compared with Tl-1. The quantitative polymerase chain reaction (qPCR) showed that the transcription of *Crt I* and *Crt B* were significantly higher in strains Sl-2 and Sl-3 than in strain Tl-1 (Figure 9B). These results indicated that the synthetic bifunctional elements designed in this study possess the ability to recruit RNA polymerase II to act on the transcriptional initiation of the target gene, and the elements can also achieve transcriptional termination. It is further demonstrated that bifunctional elements could be a promising tool for application in pathway engineering for fine regulation. In addition, the use of bifunctional elements reduces the number of required assembly fragments compared with traditional assembly methods, resulting in an increase in assembly efficiency (Figure S3). 

## 3. Methods

### 3.1. Strain, Culture Medium, and Materials

*S. cerevisiae* CEN.PK2-1C (MATa ura3-52 leu2-3, 112 trp1-289 his3DMAL2-8cSUC2) was used as the host strain. Yeast transformants in this study were selected on YPD plates (YPD plate, containing yeast extract (1%), peptone (2%), glucose (2%), agar (2%), and 500 mg/L hygromycin B). Yeast strains were cultured in YPD liquid medium (containing yeast extract (1%), peptone (2%), glucose (2%), and with corresponding antibiotics) at 30 °C and 220 rpm.

*E*. *coli* DH5a (Tiangen, Beijing, China) was used for clone and routine transformations after routine propagation in lysogeny broth (LB) medium (yeast extract (0.5%), tryptone (1%), NaCl (1%), and 100 μg/mL ampicillin). 

Oligonucleotides were synthesized by GENEWIZ (Suzhou, China). DNA polymerase Phanta Max was obtained from Vazyme (Nanjing, China). Restriction enzymes and T7 DNA ligase were purchased from New England Biolabs (NEB, Ipswich, MA, USA). The kits for plasmid extraction were from TIANGEN (Beijing, China). The kits for DNA purification were from Vazyme (Nanjing, China). The chemical reagents were all of analytical grade. A spectrophotometer (UV-5100, Shanghai Metash Instrument Co., Ltd., Shanghai, China) was used to determine the cell concentration by measuring the optical density at 600 nm (OD600). 

### 3.2. Plasmid Construction

Two fluorescence genes, eGFP and mTagBFP2 were synthesized by GENEWIZ after codon optimization (Table S4). The genes of phytoene synthase (*Crt B*, accession no.KC954270.1), phytoene desaturase (*Crt I*, accession no. AY177424.1) were cloned from the W1 strain constructed by Lixia et al. [41] Yeast promoters (CYC1 and TYS1), terminators (ADH1 and ADAL) were PCR-amplified from the S. cerevisiae CEN.PK2-1C genome. 

The plasmid used as a backbone in the construction of expression vectors in this study was pRS41H (Beijing Biosea Biotechnology Co., Ltd., Beijing, China). Specificallmand); the ligated products were then inserted into the pRS41H plasmid linearized by *Not*I and *Sac*I to form the scaffold plasmids pRS-Exp1 (Supplementary Figure S1). Five scaffold plasmids pRS-Exp1–5 were constructed according to a similar strategy. All tested promoters were cloned using primers containing sites for restriction by *Bam*HI/*Not*I and then mixed with the linearized pRS-Exp1 for characterization of the promoter activity. Similarly, all tested terminators and bifunctional elements were cloned using primers containing restriction sites for *Bam*HI/*Not*I. Using T7 ligase, they were each ligated into linearized scaffold plasmids pRS-Exp2 and pRS-Exp3. 

The construction of lycopene producing strain Sl-1 was taken as an example. First, the gene expression plasmid pRS—Bif-2*TA—*Crt B*—Bif-UAS4—*Crt I*—Bif-U7 was constructed via Gibson assembly [13]. Plasmid-based strain Sl-1 was constructed by transforming gene expression plasmid into *S. cerevisiae*. Specifically, the primer pairs for adjacent fragments were designed with flanking overlap homology sequences, as shown in Table S5. Bif-2*TA-F/Bif-2*TA-R and Bif-U7-F/Bif-U7-R, were used to amplify upstream homologous arms, Bif-2*TA, and downstream homologous arms, Bif-U7, from the plasmid pRS41H, respectively. The target gene *Crt B* and *Crt I* were amplified using primer pairs, *Crt B*-F/*Crt B*-R and *Crt I*-F/*Crt I*-R, respectively. Bif-UAS4 was amplified using the primer pair, Bif-UAS4−F/Bif-UAS4-R. Strains Sl-2, Sl-3, and Tl-1 were constructed similarly to Sl-1. The gene expression plasmid pRS—Bif-TATTTA—*Crt B*—Bif-UAS9—*Crt I*—Bif-U6, pRS—Bif-3*TA—*Crt B*—Bif-UAS5—*Crt I*—Bif-U2, and pRS—CYC1p—*Crt B*—ADH1—TYS1—*Crt I*—ADAL were ligated by a similar strategy and were transformed into *S. cerevisiae*, respectively. Sequences were verified by Sanger sequencing (Jiangsu Saisofi Biotechnology Co., Ltd., Wuxi, China).

### 3.3. Transformation of Yeast

Yeast was cultured overnight in YPD medium and then transferred into YPD medium (50 mL) with a concentration ratio of 10% and continually cultivated for 4–6 h. The yeast broth was collected in 1.5 mL centrifuge tubes, and the medium was discarded after centrifugation at 1844× *g* for 1 min. The collected cells were washed with sterile water (2 × 1 mL) and then gently resuspended in 0.1 M LiAc (1 mL). After centrifugation at 844× *g* for 5 min, the supernatant (900 mL) was discarded, and the remaining cells were used as competent cells. Then, the components, including the DNA fragments (600 ng), linearized plasmid (400 ng), 10% denatured DNA from salmon sperm (40 µL; denatured at 100 °C for 5 min), poly(ethylene glycol) (PEG3350; 480 µL, 50% *w*/*v*), and 1 M LiAc (72 µL), were added, according to the indicated sequence, to the competent cells, and fully mixed using a high-speed spiral mixer for about 1 min. After incubation for 30 min at 30 °C, DMSO (72 µL) was added to the above system. The cells were immediately heat-shocked at 42 °C for 15 min, and the supernatant was discarded after centrifugation at 1180× *g* for 1 min. Finally, 5 mM CaCl_2_ (200 µL) was used to resuspend the yeast cells. After centrifugation, the yeast cells were re-suspended in double-distilled H_2_O (100 µL) and coated on the selective medium for incubation.

### 3.4. Determination of Element Strength

The element strength is indirectly indicated by the fluorescence intensity (FI) of eGFP and mTagBFP2. Yeast cells containing the genes of eGFP and mTagBFP_2_ were cultured in YPD medium in shaking flasks at 30 °C for 12 h and then collected by centrifugation, washed with 1× PBS (pH = 7.4), and diluted to 1 × 10^6^ cell/mL for fluorescence analysis (Beckman Coulter, Kraemer Boulevard Brea, CA, USA). The green fluorescent protein eGFP (λex 488 mm, λem 507 nm) and the blue fluorescent protein mTagBFP_2_ (λex 399 mm, λem 454 nm) were detected using a FITC-A detector and PB450-A detector, respectively. Finally, the data were processed using CytExpert software (version 2.3.1.22, Beckman Coulter, Inc., Brea, CA, USA). The FI values for at least 10,000 cells in each sample were measured. The FI of eGFP and mTagBFP_2_ was obtained by normalization relative to that of the FI of *S. cerevisiae* CEN.PK2-1C with the plasmids TYS1p-eGFP-CYC1t and TYS1p-mTagBFP_2_-CYC1t, respectively. All experiments were repeated 3 times. The standard error was estimated from the square root of the estimated error variance of the quantity.

### 3.5. qPCR

Fresh samples of the cell culture were used to determine the relative expression ratio of genes via qPCR. The total RNA in the mid-log-phase yeast cells extracted was quantified by measurement of the absorbance at 260 nm with a NanoDrop 2000c spectrophotometer (Thermo Scientific, Waltham, MA, USA). Removing DNA Contamination from RNA Samples by treatment with DNase I (NEB, Ipswich, MA, USA). cDNA was transcribed from 500 ng total RNA using a high-capacity cDNA reverse transcription kit (PrimeScript RT Master Mix, TaKaRa, San Jose, CA, USA). qPCR was performed with a SYBR Premix Ex Taq II (TaKaRa) system and LightCycler^®^ 96 (Roche). *TUB1* was used as the reference gene. The primers were as follows: 5′-CCAAC TGGTT TCAAG ATCGG TA-3′ and 5′-TCCAC AGTGG CCAAT TGTGA-3′ for qPCR analysis, 5′-ACGCT GGTTC TCAAA TGCAC-3′ and 5′-AAGTG TCGTC CAA TT GAGAG-3′ for the *Crt B* gene, and 5′-GGTAA GGACG GTTTC GACAG-3′ and 5′-CGATG AATTG CAATC TCAAG-3′ for the *Crt I* gene. The PCR conditions consisted of 40 cycles at 95 °C for 5 s, 60 °C for 20 s, and 72 °C for 1 min, with a final extension at 72 °C for 10 min. The transcription levels of the function genes in recombinant *S. cerevisiae* were calculated based on the level of *TUB1* mRNA.

### 3.6. Statistical Analysis

The fluorescence was recorded by flow cytometry. Data represent the mean fluorescence intensity with error bars (Standard Error of the Mean; SEM) from three independent experiments. Statistical analysis was performed using Origin 64 software (Origin Lab Corporation, Northampton, MA, USA). Independent univariate analysis of variance (ANOVA) was used for the comparison of multiple books. The difference of 0.05 was statistically significant.

### 3.7. Samples Preparation and Metabolite Analysis by HPLC

All engineered yeasts were grown at 30 °C and 220 rpm for 36 h as a seed solution. Then, they were inoculated into unbaffled 150 mL culture flasks containing 100 mL medium and cultured in the same conditions for four days. Lycopene was extracted using the modified hot HCl/acetone method. The acetone phase containing extracted lycopene was filtered for HPLC analysis. An HPLC system (LC-2030 plus) equipped with an Inertsil ODS-3 C_18_ column (4.6 × 250 mm, 5 mm) and a photodiode array (PDA) detector was used to measure lycopene. The detection wavelength was 470 nm. The mobile phase was acetonitrile–methanol–dichloromethane solution (21:21:8) with a flow rate of 1.0 mL/min. All experiments were repeated three times. The standard error was estimated from the square root of the estimated error variance of the quantity.

## 4. Conclusions

A class of terminator–promoter bifunctional elements was designed and constructed. Three libraries of bifunctional elements were established by adjusting the TATA sequence, transcription factor binding site, and spacer region. The results show that the promoter and terminator activities of the bifunctional elements could be finely regulated by adjusting the TATA sequence, transcription factor binding site, and spacer region. The application of bifunctional elements was shown to not only improve the efficiency of pathway construction, but also increase the yield of lycopene.

## Data Availability

All data generated or analyzed during this study are included in this publication (and the Supplementary Materials).

## Supplementary Materials

The following are available online at https://www.mdpi.com/article/10.3390/ijms24129870/s1. Ref. [50] is cited in the Supplementary Materials.

## Abbreviations

upstream activating sequence	UASs
super core promoter	S_CP_
initiator	Inr
motif ten element	MTE
downstream promoter element	DPE
enhanced green fluorescent protein	eGFP
blue fluorescent protein	mTagBFP_2_
TATA box binding protein	TBP
Transcription Factor IIB	TFIIB
TFIIB recognition element	BRE
general transcription factors	GTFs
preinitiation complex	PIC
RNA polymerase	RNAP
polymerase II	Pol II
Designed core promoter (in this study)	D_CP_
Designed terminator (in this study)	D_T_
Designed promoter (in this study)	P-D_CP_
geranylgeranyl diphosphate	GGPP
