# Supplementary material for: Development of Terminator–Promoter Bifunctional Elements for Application in Saccharomyces cerevisiae Pathway Engineering
Source: Int J Mol Sci. 2023 Jun 7;24(12):9870. doi: 10.3390/ijms24129870 (PMC10298055; doi:10.3390/ijms24129870)
Supplement: Supplementary file 1 [file ijms-24-09870-s001.zip › ijms-2132034-supplementary.pdf]

Development of terminator–promoter bifunctional elements for application in *Saccharomyces cerevisiae* pathway engineering

Xiaoxia Ni†, Zhengyang Liu†, Jintang Guo, Genlin Zhang*

School of Chemistry and Chemical Engineering/Key Laboratory for Green Processing of Chemical Engineering of Xinjiang Bingtuan, Shihezi University, Shihezi, 832003, PR China

* Corresponding author: Genlin Zhang

E-mail: zhgl_food@sina.com

Tel: +86-18099937255

Supporting Information

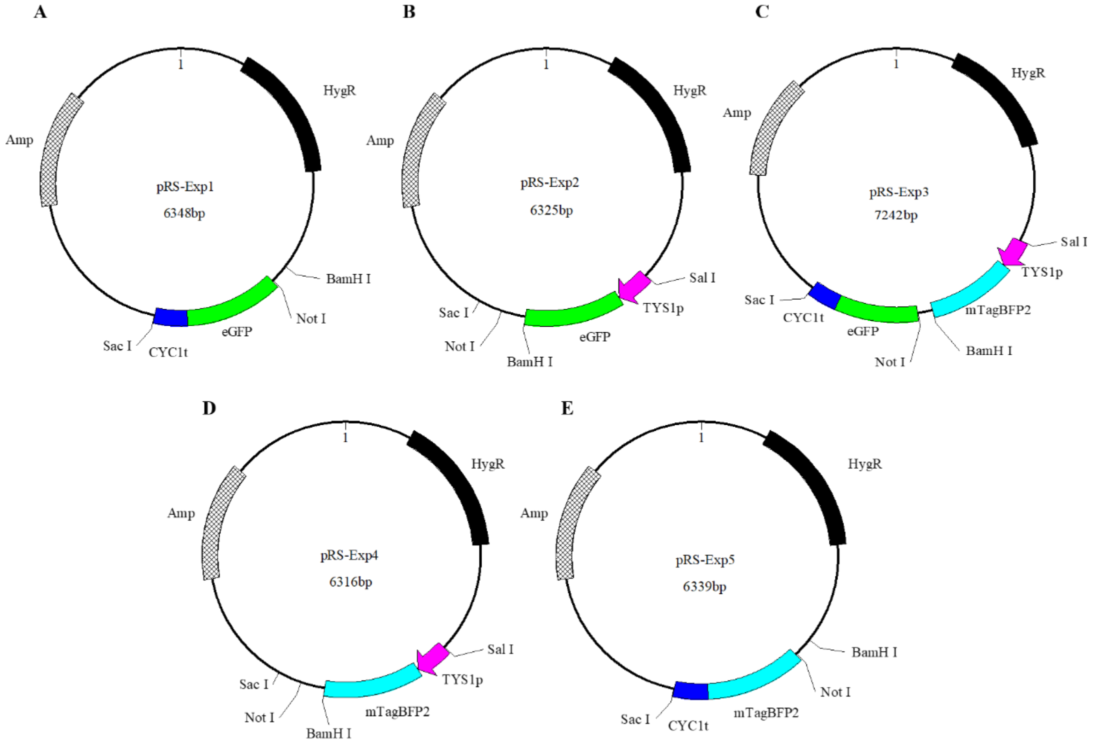

Figure S1. Evaluation of the expression levels of Promoter and terminator functional elements in *Saccharomyces cerevisiae*. (**A**) The plasmid pRS-Exp1 for validation of the promoter function was constituted by inserting the expression cassette GFP-CYC1t into the plasmid PRS41H. (**B**) Similarly, the expression cassette TYS1p-eGFP was inserted into the PRS41H to form pRS-Exp2 for validation of the terminator function. (**C**) And TYS1p-mTagBFP2-eGFP-CYC1t was constructed into the PRS41H to form an expression cassette with missing gene elements between the double transcription units, constituting the functional validation vector pRS-Exp3. All three plasmids A, B, and C were used for the validation of the gene elements designed in this paper in *Saccharomyces cerevisiae*, and the strength of the gene elements was characterized mainly by the relative fluorescence values of the corresponding

fluorescent protein expression. (**D**) The schematic diagram of plasmid pRS-Exp4, replacing eGFP in pRS-Exp1 with mTagBFP2. (**E**) The schematic diagram of plasmid pRS-Exp5, replacing eGFP in pRS-Exp2 with mTagBFP2. Constructed the elements to be validated into the corresponding plasmids using a double enzyme digestion (*Bam*HI and *Not*I restriction sites).

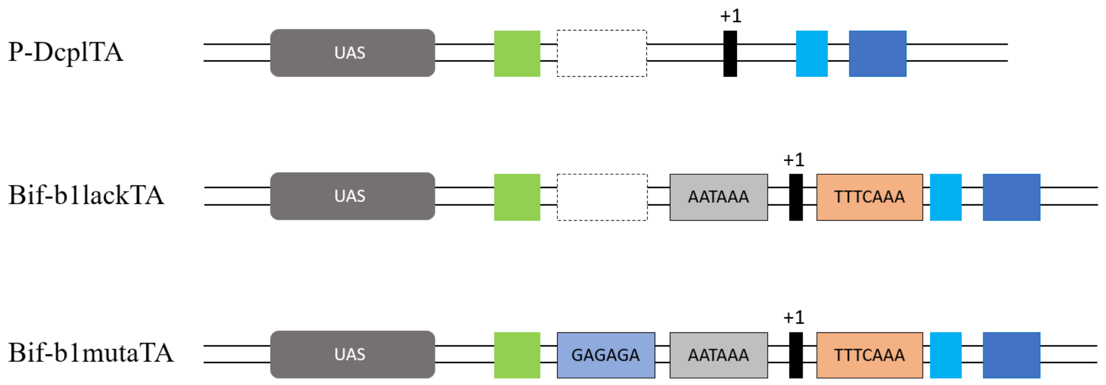

Figure S2. The Schematic illustration of deletion and mutation of TATA sequence. P-DcplTA, promoter P-Dcp lakes TATAbox. Dashed box represents deletion. Bif-b1lackTA, the terminator-promoter bifunctional element Bif-b1 lakes TATAbox. Bif-b1mutaTA, Bif-b1 mutation TATAbox sequence is GAGAGA.

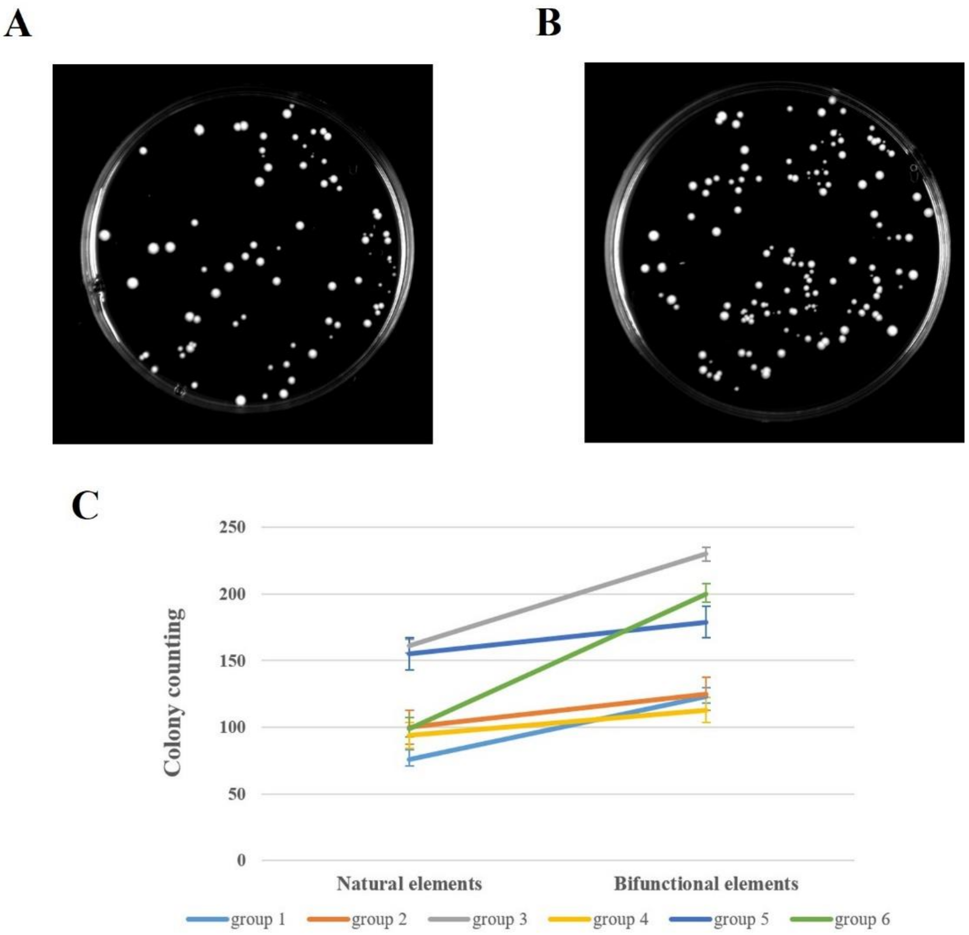

Figure S3. Plate growth of different genetic elements using Gibson assembly to construct transformants. (**A**) Plasmids used for transformation are constructed from natural genetic elements. (**B**) Plasmids used for transformation are constructed from terminator-promoter bifunctional elements. (**C**) Comparison of actual data on colony numbers of transformants constructed using natural gene elements and terminator-promoter bifunctional elements, respectively. Each plate is an independent experiment.

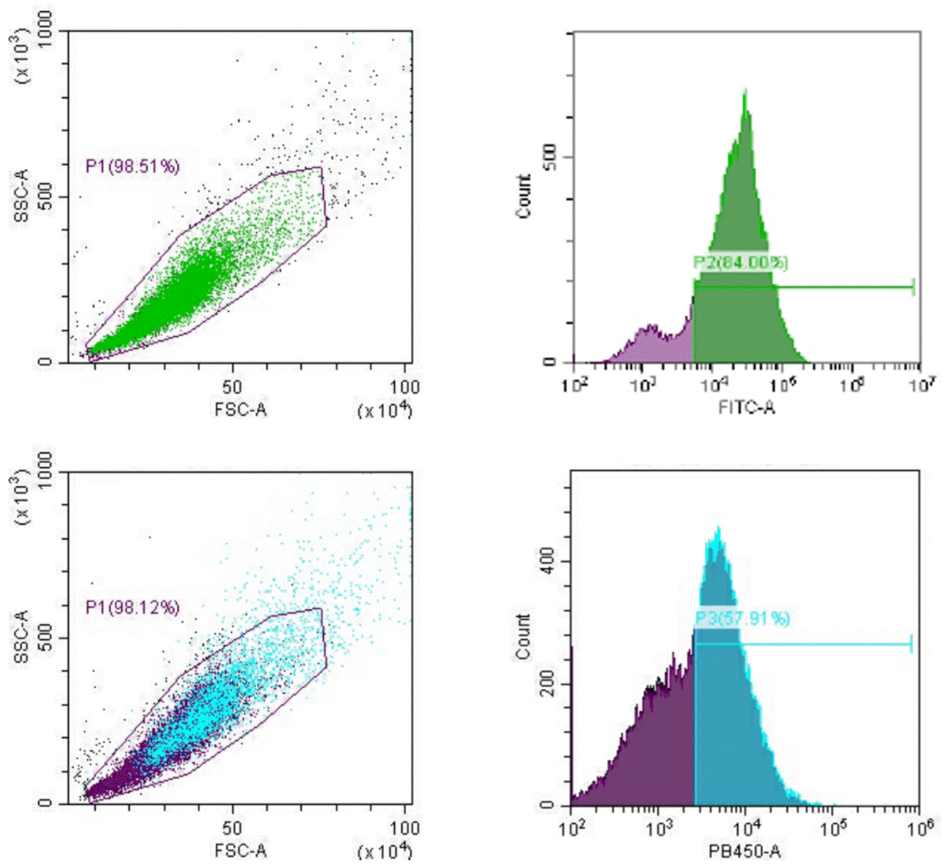

Population	Events	Total %	Parent %	Mean FITC-A	Mean PB450-A
P1	19701	98.51	98.51	39617.9	
P1	19436	98.12	98.12		13290.1
P2	16800	84.00	84.00	41770.6	
P3	11580	57.91	57.91		18165.2

Figure S4. The raw flow cytometry data of the bifunctional element Bif-b1.

Table S1 Sequences of the synthetic promoter, terminator, and bifunctional element

Name	Sequence 5′–3′
**DCP**	CTCTTGTTTTCTTCTTTTGTATAATATTCTTTTGGCATAATTCTGAGATTTGATTTACAAGTGTTTCTGTTGGACGCCTATATATAAAAGTTGTTGTTTTTTATCATTTTATCTTCCGTCGCGATCGAACGAGCCGAGCAGACGTGCCTACGGACCG
**DT**	TATATATAACAGTTCTTAATAAAGGGATAGGCCCTTTCAAA
**Bif-b1**	TATGTATATGACACCTGGACATACGTATCTGTAGACAGCTTCCTCTACCTCTTGTTTTCTTCTTTTGTATAATATTCTTTTGGCATAATTCTGAGATTTGATTTACAAGTGTTTCTGTTGGACGCCTATATATAACAGTTCTTAATAAATTTTATCATTTTATCTTCCACCTTTCAAAAGTTGTTGTTCGTCGCGATCGAACGAGCCGAGCAGACGTGCCTACGGACCG

In element DCP, the green highlighted part is the BRE, the yellow highlighted part is the TATAbox, the black highlighted part is the Inr, A is the transcription start site, the blue highlighted part is the MTE, and the purple highlighted part is DPE. The rest of the linker sequences are the predicted highest active sequences of yeast promoter 1. In element DT, The three different underlines correspond to the efficiency element, position element and Ploy (A) in Figure 1D. In the bifunctional element Bif-b1, red font and green font represent Rap1 and Gcr1 binding sites, respectively.

Table S2 Sequences of transcription factor binding sites in bifunctional elements library L2.

TFBSs	Sequence 5′–3′
**Rap1**	ACACCTGGACATAC
**Gcr1**	ACAGCTTCCTCTAC
**Gcr2**	TAGCCCCGGATTTAC
**Mig1**	GTCGAAGGAGATGCTAG

Table S3 Sequences combination of transcription factor binding sites in bifunctional elements library L2.

**bifunctional elements**	**Combination method**
**Bif-UAS1**	Rap1, Gcr1
**Bif-UAS2**	Rap1, Gcr1, Mig1
**Bif-UAS3**	Gcr1, Mig1
**Bif-UAS4**	Mig1, Rap1, Gcr1
**Bif-UAS5**	Mig1, Rap1, Gcr1, Gcr2
**Bif-UAS6**	Gcr1, Rap1
**Bif-UAS7**	Gcr1, Gcr2
**Bif-UAS8**	Gcr1, Gcr2, Mig1, Rap1
**Bif-UAS9**	Gcr1, Rap1, Gcr2, Mig1
**Bif-UAS10**	Rap1, Gcr1, Gcr2, Mig1

Table S4 Gene Sequences used in this study.

**Name**	**Sequence (5'-3')**
eGFP	ATGGTGAGCAAGGGAGAAGAACTTTTCACTGGAGTTGTCCCAATTCTTG<br>TTGAATTAGATGGTGATGTTAATGGGCACAAATTTTCTGTCAGTGGAGAG<br>GGTGAAGGTGATGCAACATACGGAAAACTTACCCTTAAATTTATTTGCAC<br>TACTGGAAAGCTTCCTGTTCCTTGGCCAACACTTGTCACTACTCTTACTTA<br>TGGTGTTCAATGCTTTTCAAGATACCCAGATCATATGAAGCGGCACGACT<br>TCTTCAAGAGCGCCATGCCTGAGGGATACGTGCAGGAGAGGACCATCTT<br>CTTCAAGGACGACGGGAACTACAAGACACGTGCTGAAGTCAAGTTTGAG<br>GGAGACACCCTCGTCAACAGAATCGAGCTTAAGGGAATCGATTTCAAGG<br>AGGACGGAAACATCCTCGGCCACAAGTTGGAATACAACTACAACTCCCA<br>CAACGTATACATCATGGCAGACAAACAAAAGAATGGAATCAAAGTTAAC<br>TTCAAAATTAGACACAACATTGAAGATGGAAGCGTTCAACTAGCAGACC<br>ATTATCAACAAAATACTCCAATTGGCGATGGCCCTGTCCTTTTACCAGACA<br>ACCATTACCTGTCCACACAATCTGCCCTTTCGAAAGATCCCAACGAAAAG<br>AGAGACCACATGGTCCTTCTTGAGTTTGTAACAGCTGCTGGGATTACACA<br>TGGCATGGATGAACTATACAAATAA
mTagBFP2	ATGGTTTCAAAGGGTGAGGAACTTATTAAAGAGAATATGCATATGAAACT<br>TTATATGGAAGGAACAGTTGATAACCATCATTTCAAGTGCACATCAGAAG<br>GAGAAGGAAAGCCGTACGAAGGAACACAAACAATGAGAATTAAAGTTG<br>TTGAAGGAGGACCTCTCCCGTTTGCTTTCGATATCCTCGCTACCTCCTTCC<br>TCTACGGGAGCAAGACTTTCATCAATCACACTCAGGGCATACCCGACTTC<br>TTCAAGCAATCATTTCCTGAAGGATTTACATGGGAAAGAGTTACAACATA<br>TGAAGATGGAGGAGTTCTTACAGCAACACAAGATACATCACTTCAAGAT<br>GGATGTCTTATTTATAACGTTAAGATACGAGGAGTTAACTTTACATCAAAC<br>GGACCTGTTATGCAGAAGAAGACCCTAGGATGGGAAGCATTTACAGAAA<br>CACTTTATCCTGCAGATGGAGGACTTGAAGGAAGAAACGATATGGCACTT<br>AAACTTGTTGGAGGATCACATCTTATTGCAAACGCAAAGACGACCTATAG<br>ATCAAAGAAGCCAGCAAAGAATTTGAAGATGCCTGGCGTATACTACGTAG

	ACTACAGGCTCGAGAGGATAAAGGAGGCAAACAACGAAACATATGTTGA<br>ACAACATGAAGTTGCAGTTGCAAGATATTGTGATCTTCCTTCAAAGTTAG<br>GCCATAAACTTAAC
Crt I	ATGGGTAAGGAACAAGACCAAGACAAGCCAACTGCTATCATCGTTGGTT<br>GTGGTATCGGTGGTATCGCTACTGCTGCTAGATTGGCTAAGGAAGGTTTC<br>CAAGTTACTGTTTTCGAAAAGAACGACTACTCTGGTGGTAGATGTTCTTT<br>GATCGAAAGAGACGGTTACAGATTCGACCAAGGTCCATCTTTGTTGTTGT<br>TGCCAGACTTGTTCAAGCAAACTTTCGAAGACTTGGGTGAAAAGATGGA<br>AGACTGGGTTGACTTGATCAAGTGTGAACCAAACTACGTTTGTCACTTCC<br>ACGACGAAGAAACTTTCACTTTGTCTACTGACATGGCTTTGTTGAAGAGA<br>GAAGTTGAAAGATTCGAAGGTAAGGACGGTTTCGACAGATTCTTGTCTTT<br>CATCCAAGAAGCTCACAGACACTACGAATTGGCTGTTGTTCACGTTTTGC<br>AAAAGAACTTCCCAGGTTTCGCTGCTTTCTTGAGATTGCAATTCATCGGT<br>CAAATCTTGGCTTTGCACCCATTCGAATCTATCTGGACTAGAGTTTGTAGA<br>TACTTCAAGACTGACAGATTGAGAAGAGTTTTCTCTTTCGCTGTTATGTAC<br>ATGGGTCAATCTCCATACTCTGCTCCAGGTACTTACTCTTTGTTGCAATAC<br>ACTGAATTGACTGAAGGTATCTGGTACCCAAGAGGTGGTTTCTGGCAAGT<br>TCCAAACACTTTGTTGCAAATCGTTAAGAGAAACAACCCATCTGCTAAGT<br>TCAACTTCAACGCTCCAGTTTCTCAAGTTTTGTTGTCTCCAGCTAAGGAC<br>AGAGCTACTGGTGTTAGATTGGAATCTGGTGAAGAACACCACGCTGACG<br>TTGTTATCGTTAACGCTGACTTGGTTTACGCTTCTGAACACTTGATCCCAG<br>ACGACGCTAGAAACAAGATCGGTCAATTGGGTGAAGTTAAGAGATCTTG<br>GTGGGCTGACTTGGTTGGTGGTAAGAAGTTGAAGGGTTCTTGTTCTTCTT<br>TGTCTTTCTACTGGTCTATGGACAGAATCGTTGACGGTTTGGGTGGTCAC<br>AACATCTTCTTGGCTGAAGACTTCAAGGGTTCTTTCGACACTATCTTCGA<br>AGAATTGGGTTTGCCAGCTGACCCATCTTTCTACGTTAACGTTCCATCTAG<br>AATCGACCCATCTGCTGCTCCAGAAGGTAAGGACGCTATCGTTATCTTGG<br>TTCCATGTGGTCACATCGACGCTTCTAACCCACAAGACTACAACAAGTTG<br>GTTGCTAGAGCTAGAAAGTTCGTTATCCACACTTTGTCTGCTAAGTTGGG<br>TTTGCCAGACTTCGAAAAGATGATCGTTGCTGAAAAGGTTCACGACGCT<br>CCATCTTGGGAAAAGGAATTCAACTTGAAGGACGGTTCTATCTTGGGTTT<br>GGCTCACAACTTCATGCAAGTTTTGGGTTTCAGACCATCTACTAGACACC<br>CAAAGTACGACAAGTTGTTCTTCGTTGGTGCTTCTACTCACCCAGGTACT<br>GGTGTTCCAATCGTTTTGGCTGGTGCTAAGTTGACTGCTAACCAAGTTTT<br>GGAATCTTTCGACAGATCTCCAGCTCCAGACCCAAACATGTCTTTGTCTG<br>TTCCATACGGTAAGCCATTGAAGTCTAACGGTACTGGTATCGACTCTCAA<br>GTTCAATTGAAGTTCATGGACTTGGAAAGATGGGTTTACTTGTTGGTTTT<br>GTTGATCGGTGCTGTTATCGCTAGATCTGTTGGTGTTTTGGCTTTCTAA
Crt B	ATGAACAACCCATCTTTGTTGAACCACGCTGTTGAAACTATGGCTGTTGG<br>TTCTAAGTCTTTCGCTACTGCTTCTAAGTTGTTCGACGCTAAGACTAGAA<br>GATCTGTTTTGATGTTGTACGCTTGGTGTAGACACTGTGACGACGTTATCG<br>ACGACCAAACTTTGGGTTTCCAAGCTAGACAACCAGCTTTGCAAACTCC<br>AGAACAAAGATTGATGCAATTGGAAATGAAGACTAGACAAGCTTACGCT<br>GGTTCTCAAATGCACGAACCAGCTTTCGCTGCTTTCCAAGAAGTTGCTAT<br>GGCTCACGACATCGCTCCAGCTTACGCTTTCGACCACTTGGAAGGTTTCG<br>CTATGGACGTTAGAGAAGCTCAATACTCTCAATTGGACGACACTTTGAGA<br>TACTGTTACCACGTTGCTGGTGTTGTTGGTTTGATGATGGCTCAAATCATG<br>GGTGTTAGAGACAACGCTACTTTGGACAGAGCTTGTGACTTGGGTTTGG<br>CTTTCCAATTGACTAACATCGCTAGAGACATCGTTGACGACGCTCACGCT<br>GGTAGATGTTACTTGCCAGCTTCTTGGTTGGAACACGAAGGTTTGAACAA<br>GGAAAACTACGCTGCTCCAGAAAACAGACAAGCTTTGTCTAGAATCGCT<br>AGAAGATTGGTTCAAGAAGCTGAACCATACTACTTGTCTGCTACTGCTGG<br>TTTGGCTGGTTTGCCATTGAGATCTGCTTGGGCTATCGCTACTGCTAAGCA<br>AGTTTACAGAAAGATCGGTGTTAAGGTTGAACAAGCTGGTCAACAAGCT<br>TGGGACCAAAGACAATCTACTACTACTCCAGAAAAGTTGACTTTGTTGTT<br>GGCTGCTTCTGGTCAAGCTTTGACTTCTAGAATGAGAGCTCACCCACCAA<br>GACCAGCTCACTTGTGGCAAAGACCATTGTAA

TYS1p	TCCTTGCGCTTACTCGAATAGGCCTCCCTAGCTATTCTTCAACCTTTCGAACCATCCATACTTCTTACTATCATAATTTTATTTTATCATGGAGGCGAGAAGGTCCTTATTCGAGCATCACTAAGAACGGAACTCGAACATTTACAAAGTAGAAAAATTTTATGAAAATTAATTGTTCTTTCTTCAGAATACAAATTAGTCATTGTCAAAAAGAGATTAGCATCCATAACCGCATACTCTAATTGACGATAACA
CYC1t	TCATGTAATTAGTTATGTCACGCTTACATTCACGCCCTCCCCCCACATCCGCTCTAACCGAAAAGGAAGGAGTTAGACAACCTGAAGTCTAGGTCCCTATTTATTTTTTTATAGTTATGTTAGTATTAAGAACGTTATTTATATTTCAAATTTTTCTTTTTTTTCTGTACAGACGCGTGTACGCATGTAACATTATACTGAAAACCTTGCTTGAGAAGGTTTTGGGACGCTCGAAGGCTTTAATTTGC
CYC1p	GCATGCATGTGCTCTGTATGTATATAAAACTCTTGTTTTCTTCTTTTCTCTAAATATTCTTTCCTTATACATTAGGACCTTTGCAGCATAAATTACTATACTTCTATAGACACACAAACACAAATACACACACTAAATTAATA
ADH1t	AGTTATAAAAAAAATAAGTGTATACAAATTTTAAAGTGACTCTTAGGTTTTAAAACGAAAATTCTTATTCTTGAGTAACTCTTTCCTGTAGGTCAGGTTGCTTTCTCAGGTATAGCATGAGGTCGCTCTTATTGACCACACCTCTACCGGCATGCCGA
ADALt	AGGGATTAATTAAATCGTAAGGAAAAATAAAATAATAGTGCTGTGATCGCATGATATTCTTCCCTGGAAGCGCCATTTTATAGCAAGAAATGTAAGTCAAGTATATTTTAACTGTATATCAACAATATAGCTCTTTTTTATGCCTTGTTGTTTTTCTTCGGGTTTTCCCCACACATTGTGTGGAGAGATAGTTATTAACAGACCGGAAAATAGCCGCCCAAGGATAAACTTTTATATAAAGGGAAGGGTAGTTGACCCAAAAAAATTGGATTCTACTTTTCCAGATTTACTTTCACCCTTTTATATTTGCTGTAGTCTGTTATGCCAATCAGGAAAGCATTTGAACAATATGTCTGTTACAGGAACTGAGATCGATAGTGATACAGCAAAAAAATATTCTTAGTGTAGATGAATTACAGAACTATGGTATTAATGCCTCAGATCTTCAAAAATTGAAGTCTGGTGGG

Table S5. Primers used for the construction of the lycopene synthesis pathway based on the synthetic bifunctional elements

**Strains**	**Primers**	**Sequence (5'-3')**
Tl-1	CYC1p-F	GATATCGAATTCCTGCAGCCCGGGGCATGCATGTGCTCTGTATG
	CYC1p-R	GTTCAACAAAGATGGGTTGTTCATTATTAATTTAGTGTGTGTATTTGTGTTTGTGTG
	CrtB-F	CACACAAACACAAATACACACACTAAATTAATAATGAACAACCCATCTTTGTTGAAC
	CrtB-R	AATTTGTATACACTTATTTTTTTTATAACTTTACAATGGTCTTTGCCACAAGTG
	ADH1t-F	CACTTGTGGCAAAGACCATTGTAAAGTTATAAAAAAAATAAGTGTATACAAATT
	ADH1t-R	CCTATTCGAGTAAGCGCAAGGATCGGCATGCCGGTAGAGG
	TYS1p-F	CCTCTACCGGCATGCCGATCCTTGCGCTTACTCGAATAGG
	TYS1p-R	GTCTTGGTCTTGTTCCTTACCCATTGTTATCGTCAATTAGAGTATGCGG
	CrtI-F	CCGCATACTCTAATTGACGATAACAATGGGTAAGGAACAAGACCAAGAC
	CrtI-R	ATTTTATTTTTCCTTACGATTTAATTAATCCCTTTAGAAAGCCAAAACACCAACAGATC
	ADALt-F	GATCTGTTGGTGTTTTGGCTTTCTAAAGGGATTAATTAAATCGTAAGGAAAAATAAAAT
	ADALt-R	CGGCCGCTCTAGAACTAGTGGATCCCCCACCAGACTTCAATTTTTG
	T1-F	GATATCGAATTCCTGCAGCCCGGGGCATGCATGTGCTCTGTATG
	T1-R	CGGCCGCTCTAGAACTAGTGGATCCCCCACCAGACTTCAATTTTTG
Sl-1	Bif-2*TA-F	CCGGGCCCCCCCTCGAGTATGTATATGACACCTGGAC
	Bif-2*TA-R	CAACAAAGATGGGTTGTTCATCGGTCCGTAGGCACGTCTGC
	Crt B-F	GCAGACGTGCCTACGGACCGATGAACAACCCATCTTTGTTG
	Crt B-R	CTCCTTCGACCATATACATATTACAATGGTCTTTGCCAC

	Bif-UAS4-F	GTGGCAAAGACCATTGTAATATGTATATGGTCGAAGGAG
	Bif-UAS4-R	GGTCTTGTTCCTTACCCATCGGTCCGTAGGCACGTC
	Crt I-F	GCAGACGTGCCTACGGACCG ATGGGTAAGGAACAAGACCAAG
	Crt I-R	CTATTCTCTCATATACATATTAGAAAGCCAAAACACCAAC
	Bif-U7-F	GTTGGTGTTTTGGCTTTCTAATATGTATATGAGAGAATAG
	Bif-U7-R	GGCTGCAGGAATTCGATATCCGGTCCGTAGGAGTAAC
	Sl-1-F	CCGGGCCCCCCCTCGAGTATGTATATGACACCTGGAC
	S1-1-R	GGCTGCAGGAATTCGATATCCGGTCCGTAGGAGTAAC
S1-2	Bif-TATTTA-F	CCGGGCCCCCCCTCGAGTATGTATATGACACCTGGAC
	Bif-TATTTA-R	CAACAAAGATGGGTTGTTCATCGGTCCGTAGGCACGTCTGC
	Crt B-F	GCAGACGTGCCTACGGACCGATGAACAACCCATCTTTGTTG
	Crt B-R	GAGGAAGCTGTCATATACATATTACAATGGTCTTTGCCAC
	Bif-UAS9-F	CTTGTGGCAAAGACCATTGTAATATGTATATGACAGCTTCCTC
	Bif-UAS9-R	CTTGGTCTTGTTCCTTACCCATCGGTCCGTAGGCACGTCTGC
	Crt I-F	GCAGACGTGCCTACGGACCGATGGGTAAGGAACAAGACCAAG
	Crt I-R	GATAGTTACCCATATACATATTAGAAAGCCAAAACACCAAC
	Bif-U6-F	GTTGGTGTTTTGGCTTTCTAATATGTATATGGGTAACTATC
	Bif-U6-R	GGCTGCAGGAATTCGATATCCGGTCCGTAGCTCTTATC
	Sl-2-F	CCGGGCCCCCCCTCGAGTATGTATATGACACCTGGAC
	Sl-2-R	GGCTGCAGGAATTCGATATCCGGTCCGTAGCTCTTATC
S1-3	Bif-3*TA-F	CCGGGCCCCCCCTCGAGTATGTATATGACACCTGGAC
	Bif-3*TA-R	CAACAAAGATGGGTTGTTCATCGGTCCGTAGGCACGTCTGC
	Crt B-F	GCAGACGTGCCTACGGACCGATGAACAACCCATCTTTGTTG
	Crt B-R	CTCCTTCGACCATATACATATTACAATGGTCTTTGCCAC
	Bif-UAS5-F	GTGGCAAAGACCATTGTAATATGTATATGGTCGAAGGAG
	Bif-UAS5-R	GGTCTTGTTCCTTACCCATCGGTCCGTAGGCACGTC
	Crt I-F	GCAGACGTGCCTACGGACCGATGGGTAAGGAACAAGACCAAG
	Crt I-R	ATCTGACTTACATATACATATTAGAAAGCCAAAACACCAAC
	Bif-U2-F	GTTGGTGTTTTGGCTTTCTAATATGTATATGTAAGTCAGAT
	Bif-U2-R	GGCTGCAGGAATTCGATATCCGGTCCGTAGAGGTTTC
	Sl-3-F	CCGGGCCCCCCCTCGAGTATGTATATGACACCTGGAC
	Sl-3-R	GGCTGCAGGAATTCGATATCCGGTCCGTAGAGGTTTC

References

1. Lubliner, S.; Keren, L.; Segal, E., Sequence features of yeast and human core promoters that are predictive of maximal promoter activity. *Nucleic Acids Res.* **2013,** *41* (11), 5569-5581.
